# Incompatibles with Corrosive Sublimate

**Published:** 1847-01

**Authors:** 


*Incompatibles with Corrosive Sublimate.*—It may be useful to know the vegetable infusions, decoctions, and tinctures, which decompose corrosive sublimate. Any pharmaceutical preparation containing one or more of the following substances will produce this effect; but the rapidity with which the decomposition takes places varies in different instances. 1st. Substances that decompose corrosive sublimate slowly, throwing down calomel: marsh mallows, bitter sweet, calumba, oak bark, sarsaparilla, quassia, gentian, resin of guaiacum. 2d. Substances that decompose corrosive sublimate instantly, forming particular mercurial compounds: opium, cinchona. The result of the decompositions produced by the first group is to diminish extremely the activity of the corrosive sublimate: thus, this medicine mixed in ordinary dose, with decoction of sarsaparilla, and administered for an indefinite period, will rarely salivate. The decomposition produced by cinchona does not, on the contrary, seem to interfere materially with the virtues of the medicine: we know that one of the most favorite modes of administering corrosive sublimate is, disolved in tincture of cinchona.—*Southern Med. Jour.*

